# Hundred year projected carbon loads and species compositions for four National Forests in the northwestern USA

**DOI:** 10.1186/s13021-020-00140-9

**Published:** 2020-03-28

**Authors:** Patrick A. Fekety, Nicholas L. Crookston, Andrew T. Hudak, Steven K. Filippelli, Jody C. Vogeler, Michael J. Falkowski

**Affiliations:** 1 Natural Resources Ecology Laboratory, Colorado State University, Fort Collins, CO 80523-1499 USA; 2 Forestry Research Consultant, Moscow, ID 83843 USA; 3 United States Forest Service, Rocky Mountain Research Station, 1221 South Main Street, Moscow, ID 83843 USA; 4 Department of Ecosystem Science and Sustainability, Colorado State University, Fort Collins, CO 80523-1476 USA

**Keywords:** Climate-FVS, Climate change, dClim rule, Forest carbon planning, Forest Inventory and Analysis (FIA), Forest Vegetation Simulator (FVS), Modeling

## Abstract

**Background:**

Forests are an important component of the global carbon balance, and climate sensitive growth and yield models are an essential tool when predicting future forest conditions. In this study, we used the dynamic climate capability of the Forest Vegetation Simulator (FVS) to simulate future (100 year) forest conditions on four National Forests in the northwestern USA: Payette National Forest (NF), Ochoco NF, Gifford Pinchot NF, and Siuslaw NF. Using Forest Inventory and Analysis field plots, aboveground carbon estimates and species compositions were simulated with Climate-FVS for the period between 2016 and 2116 under a no climate change scenario and a future climate scenario. We included a sensitivity analysis that varied calculated disturbance probabilities and the dClim rule, which is one method used by Climate-FVS to introduce climate-related mortality. The dClim rule initiates mortality when the predicted climate change at a site is greater than the change in climate associated with a predetermined shift in elevation.

**Results:**

Results of the simulations indicated the dClim rule influenced future carbon projections more than estimates of disturbance probability. Future aboveground carbon estimates increased and species composition remained stable under the no climate change scenario. The future climate scenario we tested resulted in less carbon at the end of the projections compared to the no climate change scenarios for all cases except when the dClim rule was disengaged on the Payette NF. Under the climate change scenario, species compositions shifted to climatically adapted species or early successional species.

**Conclusion:**

This research highlights the need to consider climate projections in long-term planning or future forest conditions may be unexpected. Forest managers and planners could perform similar simulations and use the results as a planning tool when analyzing climate change effects at the National Forest level.

## Background

An important component of managing forests for desired future conditions is understanding future climate regimes and their impact on forest resources. In the western USA, the United States Department of Agriculture Forest Service (USFS) manages approximately one-half of the public forests, which equates to 48.6 million ha [1] and is required by law to develop National Forest Plans to guide future management on National Forests [2]. As part of the planning process, the current USFS policy requires the consideration of changing climates in new forest plans [3].

Future climate across the western USA is predicted to lead to warmer temperatures on average, with topography strongly influencing the magnitude of temperature increase [4]. The predicted increases in temperature vary by season and location—winter and spring will see larger temperature increases in mountainous regions, whereas summer temperatures will increase as the influence of the maritime climate decreases [4]. Precipitation in the northwest USA is dominated by inter-annual fluctuations (e.g., El Niño–Southern Oscillation) and while downscaled climate projections predict slight increases in future precipitation on average (approximately 2% increase per decade), these increases are not as noteworthy as the inter-annual variations, although the timing of precipitation may change resulting in drier summers and wetter winters [5, 6]. These changes have already begun to affect forests. In the western US, large (> 400 ha) wildfire frequency and duration have increased and were associated with increased spring and summer temperatures and earlier spring snowmelt [7]. Native forest pathogens and insect populations have experienced changes in population dynamics, which directly affects forests through altered disturbance rates [8, 9]. The effects of climate change have also been linked with the decline of quaking aspen (*Populus tremuloides*) across the western USA [10].

A wealth of climate change science exists; however, translating climate change science into management actions is challenging. Studies have reported that local climate predictions are lacking, and therefore managers may not be able to design meaningful forest treatments which consider changing climates [11, 12]. In cases where local change predictions are available, forest managers may not know how to react appropriately; for example, Kemp et al. [11] reported forest managers and planners were unaware what a 3 °C temperature rise would mean for their local forest. Kemp et al. [11] also reported that forest managers believe that implementing current management techniques, such as thinnings and prescribed burning, have a greater likelihood of being accepted by foresters when managing for climate change compared to atypical practices such as assisted migration. Large-scale, in situ experiments will be fundamental to understanding the effects climate change will have on forests. One such experiment, the Spruce and Peatland Responses Under Changing Environments (SPRUCE), is manipulating temperature and carbon dioxide concentrations in an 8 ha forest ecosystem in northern Minnesota with the goal of understanding a terrestrial ecosystem's response to climate change [13]. Programs such as Adaptive Silviculture for Climate Change have been designed to bridge the gap between the science community and local forest managers and is in the process of implementing five long-term on-the-ground treatments designed to test climate change-specific silvicultural prescriptions [14]. In addition to these experiments and programs, forest and climate simulations can aid forest managers in developing treatments that account for climate change in long-term planning.

Forest and ecosystem models that use climate as an input are useful to explore the impact of various climate scenarios on future forest conditions. Many such models exist including: Landis-II [15], 3PG [16], and CLM [17] (see Öztürk el al [18]. for additional models). While these ecological process models are routinely used in research applications, they are not easily parameterized using forest inventory data and therefore are not typically used by land managers. An empirical model, the Forest Vegetation Simulator (FVS), is a popular growth and yield model used by forest managers in the USA [19, 20]. FVS is composed of sets of regional growth and yield equations known as variants. The western variants of this model include a climate extension named Climate-FVS [21, 22]. Previous studies have used Climate-FVS to investigate carbon dynamics [23–25], examine future species composition [26], identify forest trajectories on a post-wildland fire landscape [23, 27, 28], and maintain culturally important forest conditions [29, 30]. Recently, Climate-FVS results have also been used to parameterize spatially explicit climate change models that account for natural processes such as fire spread and seed dispersal [31, 32] and have been integrated with spatially explicit landscape models [33].

Climate-FVS, like any process-based model, requires certain assumptions to be made with regard to how organisms will respond to changes in their environment. Understanding the underpinnings of these assumptions and how the model results are influenced by them can both aid understanding of potential ecosystem changes and lead to model improvements. One fundamental component in Climate-FVS is species viability scores, which modifies underlying growth and mortality relationships in the FVS base model. Random forest models [34] were fit to predict species viability scores using contemporary climate data and presence-absence observations [21, 35]. Species viability scores are calculated by the Climate-FVS server [36] using downscaled climate estimates at the user-specified plot locations. A species viability score near 1.0 indicates the climate is suitable for the given species whereas a score near 0.0 indicates the species is not found in that climatic envelope. In fact, Crookston et al. [21] reported that a species is rarely present when the viability score is less than 0.5.

Climate-FVS updates mortality factors used by the base FVS model in three primary ways. First, Climate-FVS employs species viability scores to modify the maximum carrying capacity (i.e., the maximum stand density index) such that the new maximum carrying capacity reflects the tree species present on the site [21]. The carrying capacity decreases when climate favors species that occur at lower stand densities (e.g., drier species), and consequently FVS will introduce mortality if needed. Second, FVS will induce mortality if the species viability scores calculated using user-specified climate estimates suggest the species is not adapted for those climatic conditions; this procedure represents species-level mortality caused by shifting climatic envelopes [21, 22]. Third, included with the release of version 2.0 of Climate-FVS, individual tree mortality occurs if the change in climate between the model year and the tree establishment year is greater than a change in climate associated with the difference between a 300 m increase and 150 m decrease in elevation—this is known as the dClim rule [22]. The rational of the dClim rule is to account for intra-species genetic adaptation to local climate conditions. The elevation range used by the dClim rule approximates one seed zone in the western USA [22]. The dClim rule occurs regardless of the species, although it is unclear if all species are affected equally. The dClim rule is not overridden by species viability scores; therefore, the dClim rule could cause a cohort to die and be replaced by a new cohort of the same species because the species could still be considered viable in the new climate. The new cohort would be resistant to climate-induced mortality until the climate again changed enough to retrigger the dClim rule. The default behavior of Climate-FVS is to initialize the dClim rule although, its inclusion has been questioned because it was been reported to result in sudden die offs of cohorts [37], even though individual trees may vary in resistance to climate-induced mortality because of life stage or genetic adaptation [38, 39]. The combination of these three mortality pathways is responsible for climate-induced mortality.

The objective of this study is to model the potential effects of climate on forests in the northwestern USA. Specifically, we provide estimates and trajectories of aboveground forest carbon and forest composition for four National Forests in Idaho, Oregon, and Washington from 2016 to 2116. We accomplish this by using Climate-FVS to simulate future forest conditions of Forest Inventory and Analysis (FIA) field plots in four National Forests under a no climate change scenario and a climate change scenario. The effects of disturbance probabilities and the dClim rule on total carbon are also examined. These simulations demonstrate the utility of Climate-FVS as a tool for regional forest managers to incorporate into planning efforts to mitigate climate change impacts at the National Forest level.

## Methods

### Study area

**Fig. 1:**
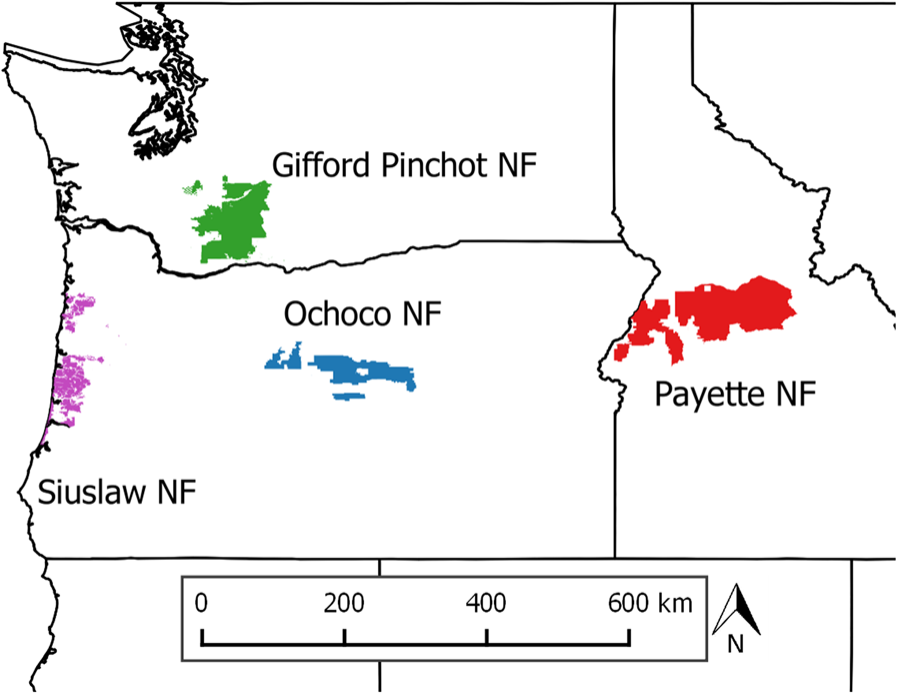
Location of National Forests used in this study

The focus of this study is four National Forests located in the northwestern USA: Payette National Forest (NF), Ochoco NF, Gifford Pinchot NF, and the Siuslaw NF (Fig. 1). We chose these forests because they represent a gradient of continental to maritime climate and disturbance regimes, which ultimately influence species composition and forest productivity (Table 1). The Payette NF located in central Idaho is a dry mixed-conifer forest with ponderosa pine (*Pinus ponderosa*) and Douglas-fir (*Pseudotsuga menziesii*) in the lower elevations and spruce-fir forests in the higher elevations. A large portion (34%) of the Payette NF is the Frank Church River of No Return Wilderness area that is excluded from timber harvest [40]. The Ochoco NF is a dry mixed conifer forest located in the rain shadow of the Cascade Range and receives the least precipitation of any forest in this study. Lower elevations support sagebrush (*Artemisia* spp.) and juniper (*Juniperus* spp.) communities and the dominant tree species are ponderosa pine, Douglas-fir, grand fir (*Abies grandis*), and western larch (*Larix occidentalis*) [41]. The Gifford Pinchot NF is on the western slope of the Cascade Range and receives ample precipitation. Major conifer species include Douglas-fir, western hemlock (*Tsuga heterophylla*), and western redcedar (*Thuja plicata*), whereas bigleaf maple (*Acer macrophyllum*) and red alder (*Alnus rubra*) are major deciduous tree species [42]. The Siuslaw NF is located in the Coast Range and its proximity to the Pacific Ocean is the primary climatic driver on the forest. Major tree species include Douglas-fir, Sitka spruce (*Picea sitchensis*), western hemlock, red alder, western redcedar, and bigleaf maple. The Siuslaw NF is very productive and produced the most timber, primarily through restoration treatments, of the four National Forests selected for this study [43].

**Table 1:** Descriptive statistics of National Forests used in this study

National Forest	State	Area (ha)	MAT (°C)	MAP (mm)	Plots used for projections	Plots with repeat measurements
Payette NF	Idaho	930,000	2.6	757	335	153
Ochoco NF	Oregon	340,000	5.9	406	343	227
Gifford Pinchot NF	Washington	550,000	5.7	2146	606	323
Siuslaw NF	Oregon	250,000	10.4	2222	308	185

*MAT* mean annual temperature, *MAP* mean annual total precipitation

The current climate of the northwestern USA (Washington, Oregon, and Idaho) transitions between maritime in western Oregon and Washington to a continental climate in the east; the Cascade Range is a major barrier separating these two regimes [44]. Mountains, which are predominantly forested, receive precipitation from orographic lift leading to wetter, more productive western slopes, and drier eastern slopes [44]. Between 1990 and 2090, mean annual temperature is expected to increase more on the eastern slope of the Cascade Range (Additional file 1: Figure S1), and mean annual precipitation (Additional file 1: Figure S2) is expected to fluctuate between 2030 and 2060 cumulating with slight regional increases for much of the Northwest with the exception of the Olympic Peninsula and the northern Cascades, which may experience large increases in precipitation.

### Data

We used data from the Forest Inventory and Analysis (FIA) [45] program to estimate current forest conditions for the study area. The USFS administers the FIA program, which provides a systematic sample of public and private forested land with 1 sample plot distributed every 2400 ha across the USA [46]. In the western USA, 10% of FIA plots in each state are measured annually, such that each plot is remeasured every 10 years. We converted FIA field measurements between 2007 and 2016 (Table 1) to FVS-readable format using FIA2FVS software to initialize the Climate-FVS simulations. We estimated disturbance probabilities and disturbance magnitude for each National Forest by querying the complete FIA database to identify field plots with repeat measurements. Field calls from the FIA database describing the disturbance type were grouped into 3 categories: harvest, fire, and stress (e.g., from insects or forest pathogens). We calculated the probability of a disturbance in a 10-year period as the proportion of plots disturbed among field plots that were remeasured. Not all disturbances are equivalent; therefore, the plot-level proportion of basal area killed by the disturbance was used as a proxy for disturbance magnitude and was represented as cumulative distribution functions. Under current management regulations, portions of National Forests may be excluded from harvesting (e.g., wilderness areas), and therefore the probability of harvest was set to zero for plots identified as reserved. The probability of disturbance and distribution of disturbance magnitudes were used to randomly initialize disturbances during the Climate-FVS simulations (described below).

### Climate-FVS simulations

FIA field plots were loaded into FVS and simulations were run using Climate-FVS under two climate scenarios—no climate change and Ensemble 6.0 (described below). The climate parameters for the no climate change scenario were climate normals for the period 1960–1990, and these values were set to be constant during the 100-year simulations. The Ensemble 6.0 is a Climate-FVS future climate scenario based on RCP6.0 models reported in the 5th assessment of the Intergovernmental Panel on Climate Change [22]. We focused on Ensemble 6.0 because it is a moderate future climate scenario, yet the predicted change in climate may allow forest managers the ability to maintain current forest conditions. Climate-ready FVS data (i.e., future climate estimates and species viability scores) for the FIA plots were obtained from the FVS-Climate Server [36] and loaded into the FVS input database. Examples of forest-wide climate values are summarized in the supplementary section (Additional file 1: Table S1).

Regeneration was simulated within Climate-FVS by planting up to 4 different species at a seedling density of 1235 trees per hectare when the stocking level fell below 40% [22]. We felt this was a conservative reforestation density that exceeds current restocking requirements. FVS can allow for natural regeneration; however, reliable natural regeneration establishment rates covering the entire study area would have been required to parameterize the model. Our reasoning for using artificial regeneration was to ensure seedlings always had the opportunity to be present. No allowance was made to ensure that a seed source was present at the plot; this effectively allowed for new species to migrate into the simulated plot. The species regenerated were limited to species currently found in the respective FVS variant, and preference was given to species climatically suited for the site at the time the regeneration was simulated.

The maximum height that a tree could grow was modified within FVS by specifying a height cap. This was done to mitigate an unfortunate behavior of the base FVS model to sometimes simulate unreasonable tree heights. Tree height caps are often added by FVS users but only take effect when trees approach or surpass the cap. The height caps we used ensure trees would not grow taller than observed within a given ecoregion. The maximum height for each tree species located in a specific variant was queried from field measured tree heights in the FIA database. These maximum heights became the preliminary values for the FVS height cap. The maximum measured heights were reviewed to determine if the heights appeared to be morphologically reasonable. In cases with abnormally low maximum heights, the height cap was replaced with a more realistic value from a geographically neighboring variant.

A sensitivity analysis was performed on model outputs by varying the disturbance probabilities and the effect of the dClim rule. In addition to the calculated disturbance probabilities (herein referred to as base disturbance level), simulations were run with the disturbance probabilities doubled, halved, and set to zero (i.e., no disturbance). Six climate metrics returned by the climate server enforce the dClim rule [22] and these values were also doubled (referred to as dClim 2.0), halved (referred to as dClim 0.5), and excluded (which disables the dClim rule; referred to as dClim Off). It is important to note that halving the dClim values increases climate-related mortality, implying species survival is tuned to a narrow climatic range; conversely doubling dClim values decreases climate-related mortality. We performed a sensitivity analysis on a range of dClim values to investigate the importance of this parameter though some values, such as dClim 0.5, may not be realistic. Excluding dClim values still allows Climate-FVS to induce climate-related mortality solely through calculated species viability scores.

We focused on two model response variables: the aboveground carbon pool representing carbon found in living and standing dead trees, and species composition. Plot-level carbon was calculated using the Fire and Fuels Extension to FVS, which uses the National Volume Estimator Library [47] allometric volume equations and species-specific density estimates to calculate carbon in the bole and regionally calibrated allometric equations to estimate carbon stored in the branches and leaves [48]. The total carbon pool for each National Forest was calculated by multiplying plot-level carbon by the plot expansion factor calculated by FIA2FVS. Similarly, species composition was calculated by multiplying the species-level basal area by the field plot expansion factor. Climate-FVS was run 10 times for each dClim—disturbance combination to obtain estimates of variation associated with model predictions resulting from disturbances being randomly assigned to the simulated plots.

## Results

**Fig. 2:**
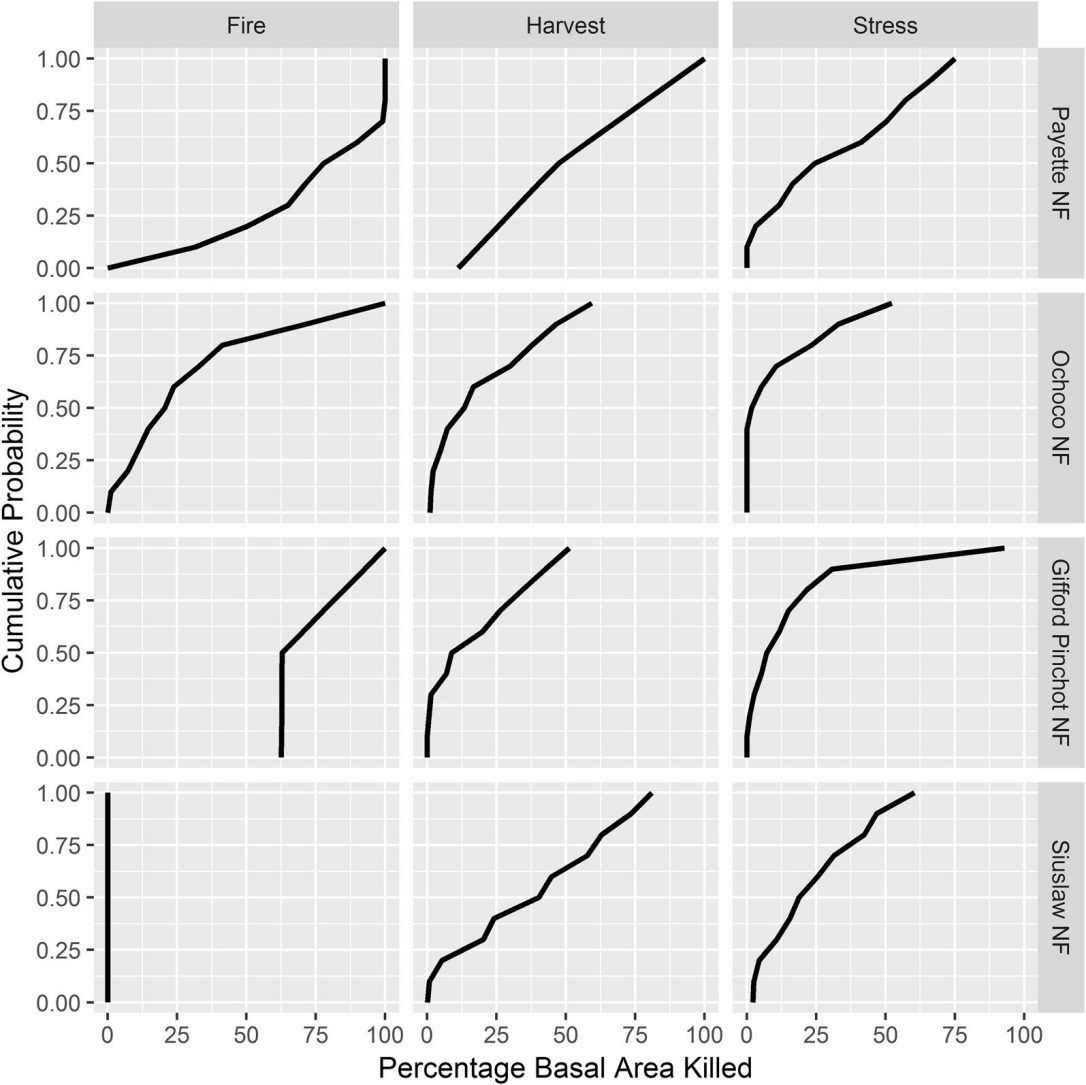
Cumulative distribution functions describing proportion of plot-level basal area killed by a given disturbance on the National Forests in this study. These cumulative distribution functions were used by Climate-FVS to simulate the disturbance magnitude. Note that the Siuslaw NF did not experience any fire events and therefore the cumulative distribution function is represented as a vertical line at 0% basal area killed

Disturbance probabilities calculated from all repeat measurements in the FIA database highlight the different disturbance regimes among the four National Forests (Table 2). However, probability of disturbance is only one component used to describe the disturbance in these FVS simulations and must be viewed alongside the calculated disturbance magnitudes (Fig. 2). For example, the Payette NF had the lowest probability of experiencing a harvest (p = 0.020; Table 2); however, when a disturbance was simulated, the proportion of basal area removed was large (Fig. 2). In fact, the Payette NF was the only forest in this study that observed a harvest where all trees were removed from the FIA plot. Similarly, the probability of a fire occurring on the Gifford Pinchot NF is low (p = 0.012; Table 2), yet when a fire occurs a large proportion of the trees are affected (Fig. 2).

**Table 2:** Base 10-year disturbance probabilities calculated from repeat FIA measurements on individual national forests that were used in the Climate-FVS simulations (i.e., proportion of plots disturbed among field plots that were remeasured)

National Forest	Harvest	Fire	Stress
Payette NF	0.020	0.144	0.078
Ochoco NF	0.132	0.093	0.335
Gifford Pinchot NF	0.050	0.012	0.149
Siuslaw NF	0.119	0.000	0.141

These probabilities were used in Climate-FVS simulations when determining if a plot was selected to be disturbed

Harvest disturbances included commercial harvesting, thinning, fuel reductions, and any tree removed by a human from the FIA plot; therefore, all harvest disturbances will result in trees being removed from the simulated FIA plot. On the other hand, fire and stress events do not always result in tree mortality, which is reflected in the fire and stress magnitude curves (Fig. 2), where the percentage basal area killed is zero. For example, 40% of the stress events on the Ochoco NF resulted in no trees being killed (Fig. 2). Common stressors in the FIA database included pathogens and insects, which are host-species specific and a function of stand characteristics; although possible, stress disturbances never resulted in complete mortality on an FIA plot used in this study. The calculated probability of fire decreased along an east–west gradient, culminating with no observed fires on FIA plots in the Siuslaw NF.

**Fig. 3:**
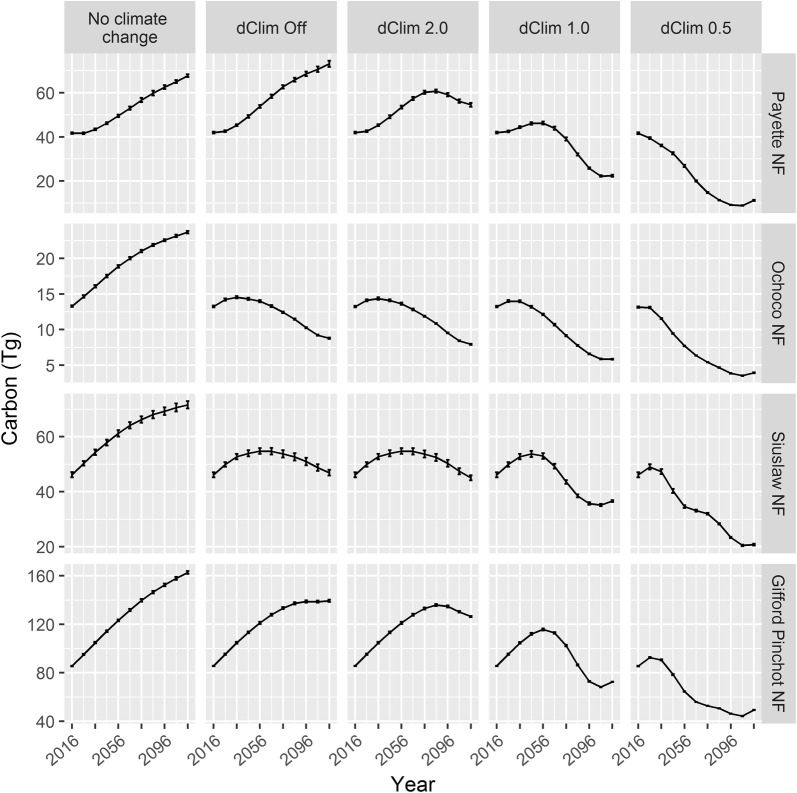
Above ground carbon estimates using base disturbance level for simulations under no climate change and Ensemble 6.0 climate scenarios at varying levels of dClim. Error bars display the 95% prediction interval

**Fig. 4:**
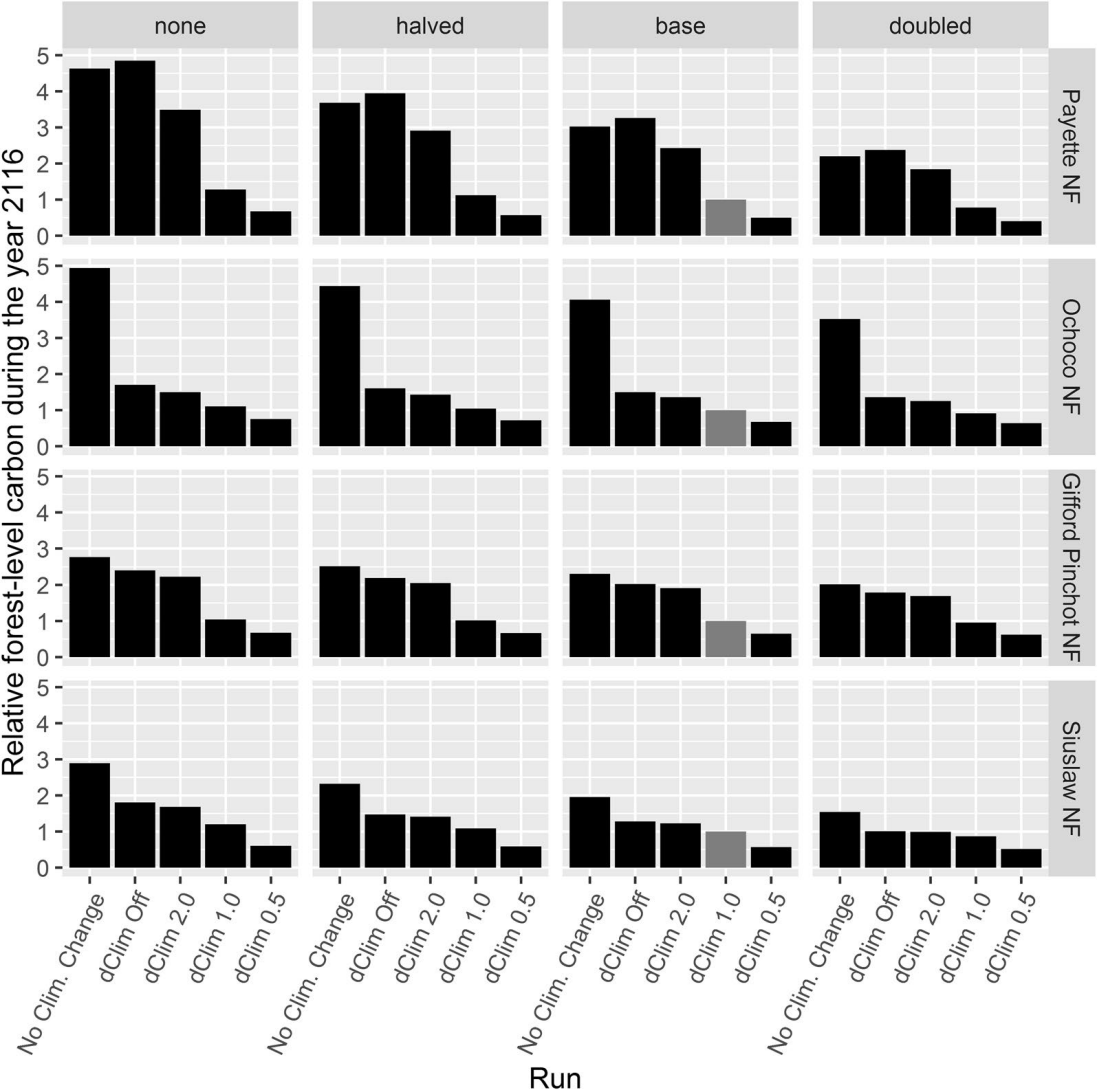
The ratio of simulated total forest-level carbon during the year 2116 calculated under various disturbance levels and dClim levels compared to carbon estimates calculated with default climate settings (dClim 1.0) and base disturbance level (shown in gray)

The dClim rule influenced future carbon values more than the stochastic effect of disturbance assignment in Climate-FVS (Fig. 3) and more than the disturbance probabilities (Fig. 4). Varying disturbance probabilities gauged how influential disturbance estimates were to the overall projections. For example, the Gifford Pinchot NF, which had relatively low base levels of disturbance probability and magnitude, was least sensitive to disturbance probability, and doubling the disturbance probability only decreased the end of simulation carbon projections by 4%, whereas disabling the dClim rule more than doubled the amount of carbon in the year 2116 (Fig. 4). Because the dClim rule had a larger influence on future carbon estimates, the remaining results will focus on the model runs that used the base disturbance level.

Carbon projections under the no climate change scenario increased across the forests highlighted in this study, assuming that current management practices and disturbance rates are maintained. Over the 100-year simulation, the carbon pool increased 62%, 78%, 90%, and 55% compared to 2016 values for the Payette NF, Ochoco NF, Gifford Pinchot NF, and Siuslaw NF, respectively. Introducing the Ensemble 6.0 climate change scenarios generally resulted in a short-term increase in forest-level carbon estimates followed by a decrease; the exceptions being Payette NF in the dClim Off simulations when carbon continued to increase, and the Payette NF and Ochoco NF dClim 0.5 simulations which experienced immediate decreases in carbon. Comparing the dClim Off simulations to the runs where the dClim rule was enabled allowed for investigation of the effect of this parameter. Situations where carbon estimates from dClim 2.0 tracked the dClim Off trajectories, e.g. Siuslaw NF (Fig. 3), indicate the change in climate was not large enough to invoke the dClim rule on the majority of plots. Carbon estimates generated with dClim 1.0, the Climate-FVS default setting, experienced a short-lived increase peaking between 2030 and 2060, when climate change became severe enough that the dClim rule was enforced. The most severe implementation of the dClim rule (dClim 0.5) resulted in the largest decreases in carbons pool over the 100-year simulations: carbon decreased 73%, 70%, 42%, and 55% for the Payette NF, Ochoco NF, Gifford Pinchot NF, and Siuslaw NF, respectively.

**Fig. 5:**
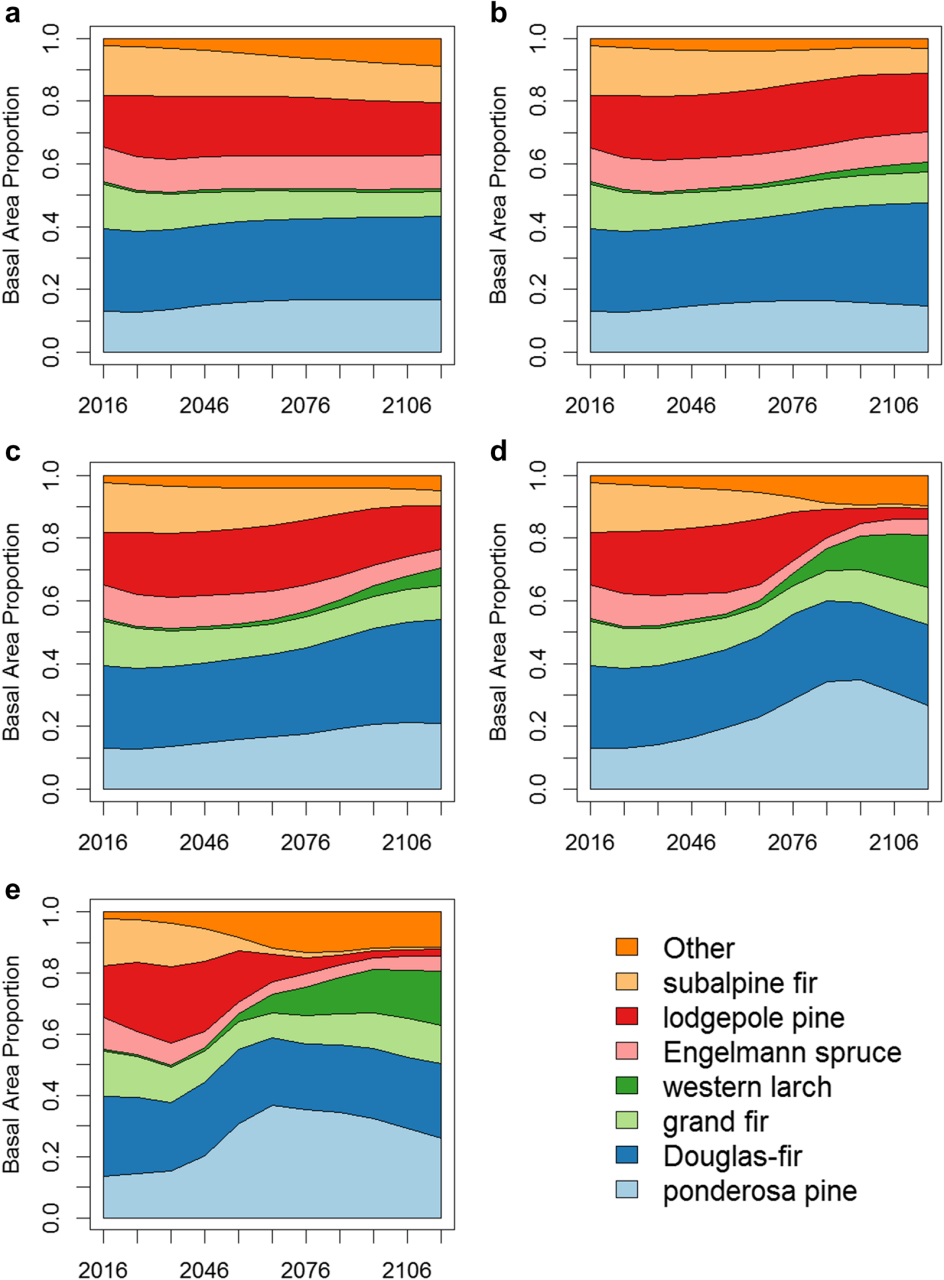
Basal area proportions for select tree species on the Payette National Forest simulated with the base disturbance level and **a** no climate change, **b** dClim Off, **c** dClim 2.0, **d** dClim 1.0 (default setting), and **e** dClim 0.5

**Fig. 6:**
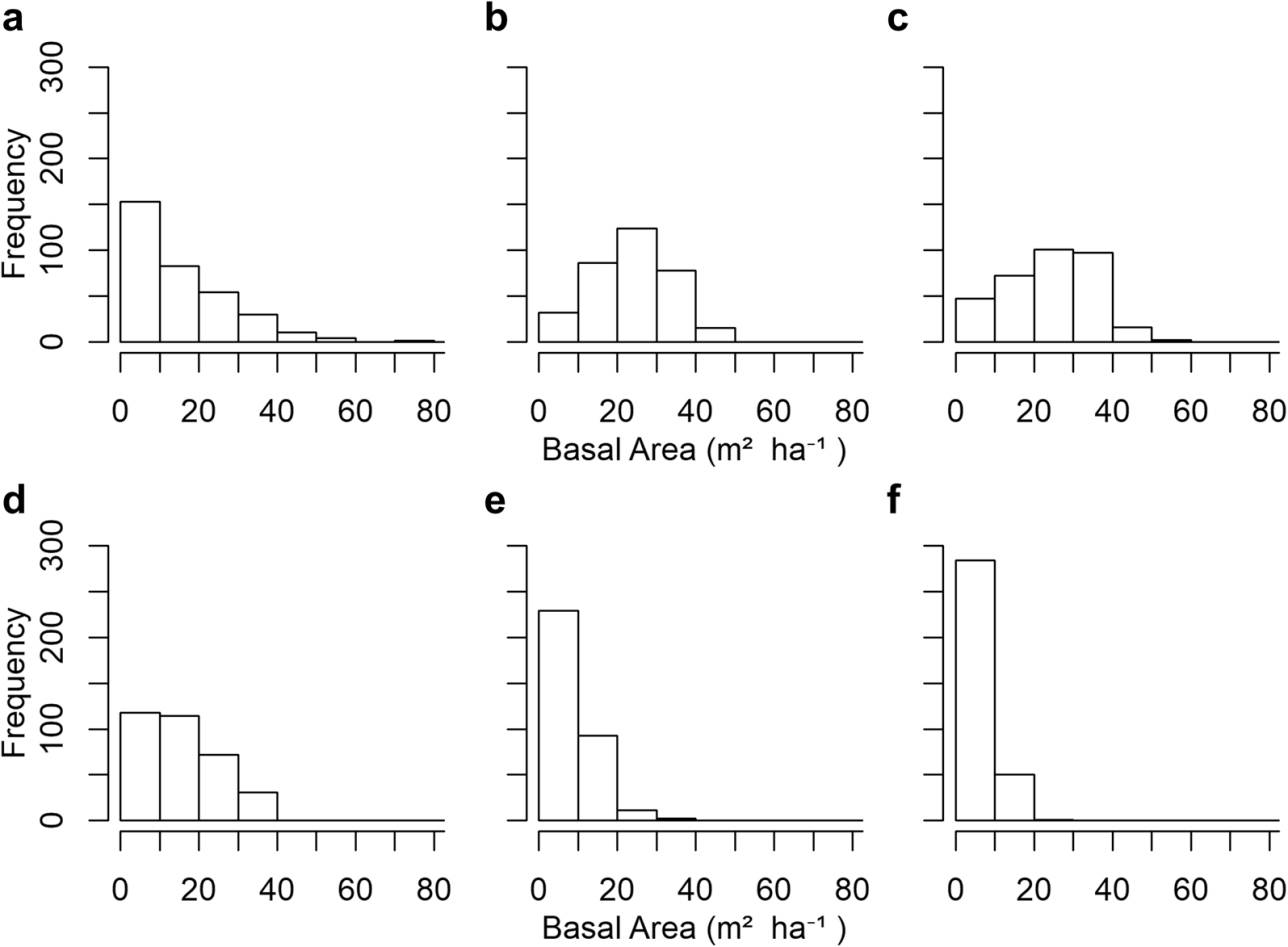
Payette National Forest plot-level basal area distribution simulated using the base disturbance level for **a** current conditions (year = 2016); **b** future conditions under no climate change (year = 2116); **c** future conditions under dClim 2.0 (year = 2116); **d** future conditions under dClim Off (year = 2116); **e** future conditions under dClim 1.0 (year = 2116); **f** future conditions under dClim 0.5 (year = 2116)

Under the no climate change scenario and dClim 2.0, species composition remained relatively constant (Fig. 5, Additional file 1: Figures S3–S5) and plot-level basal area increased (Fig. 6, Additional file 1: Figures S6–S8). Climate-FVS simulations predicted that forest-level species composition will shift under the Ensemble 6.0 climate (Fig. 5, Additional file 1: Figures S3–S5) and plot-level basal area will decrease (Fig. 6, Additional file 1: Figures S6–S8). Species adapted to drier conditions may become more predominant on the National Forests in this study area, for example western juniper and ponderosa pine will contribute a larger proportion of the basal area on the Ochoco NF at the expense of grand fir and western larch [Additional file 1: Figure S3(B–E)]. Some species, such as grand fir on the Ochoco NF [Aditional file 1: Figure S3(B–E)] and western hemlock on the Siuslaw NF [Additional file 1: Figure S5(B–E)], might even be extirpated. Red alder and bigleaf maple are early successional species, and the simulations suggest larger proportions of these species on the Gifford Pinchot NF and Siuslaw NF [(Additional file 1: Figures S4(D, E), S5(B–E)] in 2116.

## Discussion

### Carbon trends

Carbon projections differ drastically between simulations using no climate change and the Ensemble 6.0 climate change scenarios (Fig. 3), and the dClim rule is a major contributing factor. The dClim Off scenarios represent anticipated species-level changes to new climatic conditions whereas initiating the dClim rule also incorporates changes at the level of the individual tree. The dClim rule uses the change in local climate associated with a shift in elevation as a proxy for adaptiveness. This study demonstrated that varying the influence of the dClim rule can have a significant impact on future carbon and species composition estimates; similar results were reported in Diaz et al. [37] and Bugmann et al. [49]. Users of Climate-FVS must inspect results to ensure they are reasonable and consider the influence of parameterization, particularly with regard to the dClim rule.

Results from the dClim Off simulations reflect mortality caused by changes in the maximum carrying capacity and future climate being outside species-specific climatic envelopes calculated from the species viability scores. The future climate for the Payette NF and Gifford Pinchot NF (under Ensemble 6.0 with dClim Off) will not experience large shifts in the location of suitable climatic envelopes as indicated by the continual increase in carbon and the stable species composition over the 100-year simulation. However, we can infer that major tree species on Ochoco NF and Siuslaw NF will be outside their climatic envelopes in 2036 and 2066, respectively, as seen by the decrease in carbon estimates, change in species composition, and plot-level basal area shifting to lower densities.

The dClim rule was responsible for sudden drops in carbon, and the timing of these decreases relates to when the change in climate may affect individual trees as opposed to the entire species, as was the case in the dClim Off scenarios. As the magnitude of the dClim rule increases, carbon loss occurs sooner, and the total amount of carbon lost is also greater. It is not known to what degree, if any, and how fast this mortality will occur in real forests though existing research indicates that climate-induced mortality from drought, wildfire, and insect epidemics could be widespread [50, 51]. Climate-induced mortality functions vary among climate sensitive vegetation models, and a recent study found Climate-FVS was noted to be more sensitive to climate-induced mortality than non-climate induced mortality (e.g., mortality due to increasing stand density) among the models tested [49]. Toward the end of the simulations, after the dClim rule affected tree mortality rates, carbon projections stabilized or began to increase. The period of decreasing total carbon can be interpreted as when climate-induced mortality was occurring faster than the simulated forest could respond through regeneration of climatically-adapted individuals. Trees regenerated by Climate-FVS will be better suited for the expected future site conditions and will not be impacted by the dClim rule until climate again changes enough to reinitiate this mortality mechanism. These newly regenerated trees result in new forest communities as seen in shifting species compositions (Fig. 5). The model simulations in this study concluded in 2116, before these newly established stands had an opportunity to reach full maturity and contribute substantially to total carbon estimates, though small increases in carbon towards the end of simulations appear to reflect this pattern of mortality followed by regeneration. We could have chosen to run the simulations for a longer time period (e.g., 300 years) to investigate if future carbon values reached levels greater than initial conditions; however, a simulation study of such a length would not have been prudent because there is uncertainty surrounding both future climate conditions and how the forest will respond to the future climate. If the assumptions underlying the dClim rule are correct, then forests may indeed show this pattern of change with a rapid decrease in total carbon followed by a gradual increase as the forest regenerates with a better-adapted species composition.

The dClim rule affects all species equally and incorporating species-specific mortality responses to a changing climate would improve predictions. Incorporating species-specific information on genetic diversity and local adaptation [52, 53] into the dClim rule may be one route to improve the dClim rule. For example, Johnson et al. [54] reported research conducted by Rehfeldt [55] that showed genetic differences of Douglas-fir were identified at elevational differences larger than 200 m, whereas genetic differences in ponderosa pine did not occur until 450 m. A genetic difference between two trees of the same species may indicate that one individual tree is not perfectly tuned for a given site; while changes in climatic conditions associated with these elevation ranges are expected to affect the growth characteristics of individual trees with genetic differences [56], the magnitude of climatic change before mortality occurs remains unknown. Additionally, trees may respond differently to changes in climate within a species based on their size and age [57, 58], such as through drought-induced mortality caused by carbon starvation or hydraulic failure [59], which the dClim rule does not consider. This study highlighted the effect of the dClim rule, and Climate-FVS users should consider varying the dClim values to determine the effect on their response variables.

### Assumptions and limitations

Many studies using Climate-FVS have examined numerous future climate scenarios [23–25, 27–29] and found that greater deviation from current climate increases the likelihood of changes in forest structure and composition. With that in mind, we chose to investigate a moderate future climate scenario, i.e. Ensemble 6.0, and compare it to no climate change. We acknowledge there is uncertainty regarding the future climate, especially relating to what actions humans will take to combat climate change. For example, the Intergovernmental Panel on Climate Change predicts a mean temperature increase of 2.2 °C (range: 1.4–3.1 °C increase) in global mean temperature in 2100 compared to the 1986–2005 period for the RCP6.0 climate scenario [60]. Increases in mean temperature predicted by the Climate-FVS server and consequently used by Climate-FVS for the National Forests in this study were within this range (Additional file 1: Table S1).

Climate-FVS, like all models, operates under numerous assumptions and other studies have discussed the limitations of FVS and Climate-FVS [20, 24, 26], and it is important to note that Climate-FVS cannot be validated at the 100-year time scales used in this study. A major focus of this study is the dClim rule used by Climate-FVS, which is a model component that has not, and cannot, be validated. Empirically-based models, such as FVS, were calibrated using historical data and the underlying assumptions associated with that calibration might not be valid under future climates [49]. Only further research on individual tree mortality along dynamic climatic gradients can support the assumptions underlying the dClim rule and assist in tuning this factor appropriately. A major assumption in this study is that disturbance probabilities included in these scenarios are reasonable and they will remain constant over the 100 year simulations; however, it is possible to parametrize Climate-FVS such that disturbance probabilities are dynamic. Disturbance estimates used in this study are based on a relatively small number of observations, collected over a narrow time frame. Indeed, all the disturbances modeled in this study operate on time scales longer than the data used to calculate disturbance probabilities. Mortality rates for fire and stress disturbances used in the Climate-FVS simulations are similar to those reported by [61], which were calculated for all USFS land in Oregon and Washington, USA. The field data used to calculate disturbance probabilities in this study did not indicate any fire on the Siuslaw NF demonstrating a limitation of the FIA source data used in this study; however, [61] provided mortality estimates of trees disturbed by fire in wet forest types, which would include National Forests similar to the Siuslaw NF. As more data are collected by the FIA program, more realistic disturbance rates and magnitudes could be calculated. However, doubling the disturbance probabilities had less of an effect than the dClim rule on 2116 simulated carbon estimates and species compositions.

Climate related mortality is difficult to predict because often mortality is caused by an extreme event (e.g., drought) or external stress agents taking advantage of a weakened tree (e.g., bark beetle). Mortality rates in western forests have increased since the 1970s and have been attributed to climate change [62]. Long term monitoring plots, such as FIA field plots, are needed to monitor mortality. As future climate is realized, FIA data should be available to determine if the mortality rates and carbon trajectories used in this study are realistic.

Finally, Climate-FVS is only parameterized for variants in the western USA, and forests in this region are primarily found in mountainous ecosystems. The dClim rule as currently implemented might not be appropriate in other regions, for example the Lake States or southern USA, which have smaller elevation gradients across the landscape. Parameterizing Climate-FVS for variants outside the western USA would require modifications to the dClim rule. In addition to elevation, equivalent changes in latitude and longitude [63, 64] could be incorporated into the dClim parameters. Additionally, species-specific dClim rules could be introduced that better represent the climatic drivers for species within and outside the western USA. Ultimately, a better understanding of how trees respond to dynamic climatic conditions is needed to improve process-based forest simulation models for providing more reliable estimates of future carbon and species composition under different climate change scenarios.

### Options for future management

There is strong evidence that future climate will not be the same as past climate, and forest managers are advised to consider climate when planning future activities, such as identifying seed sources for tree planting. In fact, Gray and Hamann [65] concluded that major western USA tree species are currently ill-adapted for their current locations because climate has already changed. Having clear management goals is necessary to ensure that future forests are managed appropriately. Here we illustrate that Climate-FVS is one tool that managers can use to understand how climate change may affect forests and provide insight such that silvicultural treatments are developed accordingly.

Specific advice to managers will depend on the degree of realized climate change and management goals of the individual forest. One area of consideration for managers is post-disturbance regeneration because trees are particularly vulnerable in the seedling stage [65, 66]. The dominant conifers in the study region have long life spans (> 100 years) and can take a long time to reach sexual maturity; it is likely that conifers in the northwestern USA will be regenerating in a climate that is markedly different than the present [66]. Therefore, the realized rate of climate change will determine if natural regeneration will be sufficient to provide climatically-adapted offspring. Gary and Hamann [65] advocate planting seedlings adapted to short-term climate projections, and species viability scores used in Climate-FVS could guide species selection. This strategy would be best to ensure seedling survival; however, long-term productivity of the trees may decrease as climate continues to change. Additionally, genetic diversity should be increased by planting trees from low elevations and lower latitudes. St Clair and Howe [67] suggest seed sources may need to be moved 405–1130 m higher in elevation or 200–540 km north. In some locations, new seed transfer guidelines and assisted migration might be needed [68].

Managers could focus on density management activities for existing stands as another tactic to plan for a changing climate. For example, the Payette NF and Ochoco NF had the greatest probability of fire, and thinning stands can decrease fire severity and increase likelihood of survival [66, 69]. Reducing stand density also reduces drought stress by reducing competition among the remaining trees [66]. Managers should consider climate suitability when selecting management actions that include fuel reduction treatments and thinnings that remove species that may be climatically suited for the site. Climate-FVS simulations predict western larch and grand fir may be extirpated from portions of the Ochoco NF if future climatic envelopes shift outside these species' current ranges. Managers will need to determine if it is practical to preserve these species on the forest, or if the remaining Douglas-fir and ponderosa pine can provide comparable ecosystem services. Any density management operation should ensure species diversity is maintained. Priority placement of management activities is required because it would be impractical to treat every hectare in these National Forests. A successful management strategy must anticipate future conditions, not mimic past conditions.

## Conclusions

This study demonstrates the importance of considering future climate when performing long-term forest planning. Model simulations using Climate-FVS that failed to consider climate change, as demonstrated in the no climate change scenario, resulted in increasing carbon stocks and a consistent species composition for the National Forests in this study. Including a climate change scenario in the modeling framework resulted in decreased forest carbon stocks as climatically maladapted species died, and the species compositions shifted toward primarily drought tolerant species (Payette NF and Ochoco NF), or early successional species (Gifford Pinchot NF and Siuslaw NF). This study demonstrated a tool available for forest managers and planners that considers climate and hopefully encourages proactive steps to prepare for climate change. Users of Climate-FVS, along with other climate sensitive models, are encouraged to understand mortality mechanisms to ensure mortality estimates are appropriate for a given ecosystem.

The range of results associated with varying the parameters associated with the dClim rule suggests that forest managers will need to monitor climate-related mortality to determine actual carbon trajectories and species composition while allowing for enough time to adjust management strategies accordingly. The uncertainty surrounding the disturbance probabilities is minimal compared to how individual tree species will react to future climates. Climate-FVS simulations investigating potential changes in carbon trajectories and future species composition can provide insight to forest managers and planners to lessen the probability of being caught unprepared by expected climate change effects.

## Supplementary information

**Additional File 1.** Additional figures and table.

## Data Availability

FIA field data are available online through the USFS FIA website (https://apps.fs.usda.gov/fia/datamart/CSV/datamart_csv.html). Climate-FVS software is available through the USFS website (https://www.fs.fed.us/fvs/software/index.shtml). Plot-level climate estimates and species viability scores can be downloaded from the Climate-FVS server hosted by the University of Virginia Polytechnic Institute and State University (https://charcoal.cnre.vt.edu/climate/customData/fvs_data.php).

## Abbreviations

BA: Basal area
FIA: Forest Inventory and Analysis
FVS: Forest Vegetation Simulator
ha: Hectare
MAP: Mean annual precipitation
MAT: Mean annual temperature
m: Meter
mm: Millimeter
NF: National Forest
Tg: Teragram
USA: United States of America
USFS: United States Forest Service
