# Supplementary material for: Hundred year projected carbon loads and species compositions for four National Forests in the northwestern USA
Source: Carbon Balance Manag. 2020 Mar 28;15:5. doi: 10.1186/s13021-020-00140-9 (PMC7227189; doi:10.1186/s13021-020-00140-9)
Supplement: Supplementary file 1 — Additional File 1. Additional figures and table. [file 13021_2020_140_MOESM1_ESM.docx]

**Additional File 1: Additional Figures and Table**

Table S1 – Median current and future MAT and MAP values for the national forests in this study.

Figure S1 – Map of mean annual temperature (MAT) and predicted changes in MAT across the Pacific Northwest USA.

Figure S2 – Map of mean annual Precipitation (MAP) and predicted changes in MAP across the Pacific Northwest USA.

Figure 3S – Simulated results of basal area proportions for select tree species on the Ochoco National Forest.

Figure 4S – Simulated results of basal area proportions for select tree species on the Gifford Pinchot National Forest.

Figure 5S – Simulated results of basal area proportions for select tree species on the Siuslaw National Forest.

Figure S6 – Current and future estimates of plot-level basal area distribution simulated using different levels of the dClim rule on the Ochoco National Forest.

Figure S7 – Current and future estimates of plot-level basal area distribution simulated using different levels of the dClim rule on the Gifford Pinchot National Forest.

Figure S8 – Current and future estimates of plot-level basal area distribution simulated using different levels of the dClim rule on the Siuslaw National Forest.

Table S1. Climate normals for year 1990 and projected climate mean annual temperature and mean annual precipitation for years 2030, 2060, and 2090. Data summaries calculated as the median of 1 arc minute rasters returned by the Climate-FVS server [1] using the Ensemble 6.0 climate scenario.

|  | MAT (°C) | | | | MAP (mm) | | | |
| --- | --- | --- | --- | --- | --- | --- | --- | --- |
|  | Year |  |  |  | Year |  |  |  |
| National Forest | 1990 | 2030 | 2060 | 2090 | 1990 | 2030 | 2060 | 2090 |
| Payette NF | 2.6 | 4.1 | 5.2 | 6.6 | 757 | 766 | 765 | 806 |
| Ochoco NF | 5.9 | 7.2 | 8.3 | 9.6 | 406 | 406 | 369 | 417 |
| Gifford Pinchot NF | 5.7 | 7.0 | 8.1 | 9.3 | 2146 | 2163 | 2164 | 2267 |
| Siuslaw NF | 10.4 | 11.4 | 12.3 | 13.3 | 2222 | 2192 | 2187 | 2288 |
| Note: MAT – mean annual temperature | | | | | | | | |
| MAP – Mean annual total precipitation | | | | | | | | |


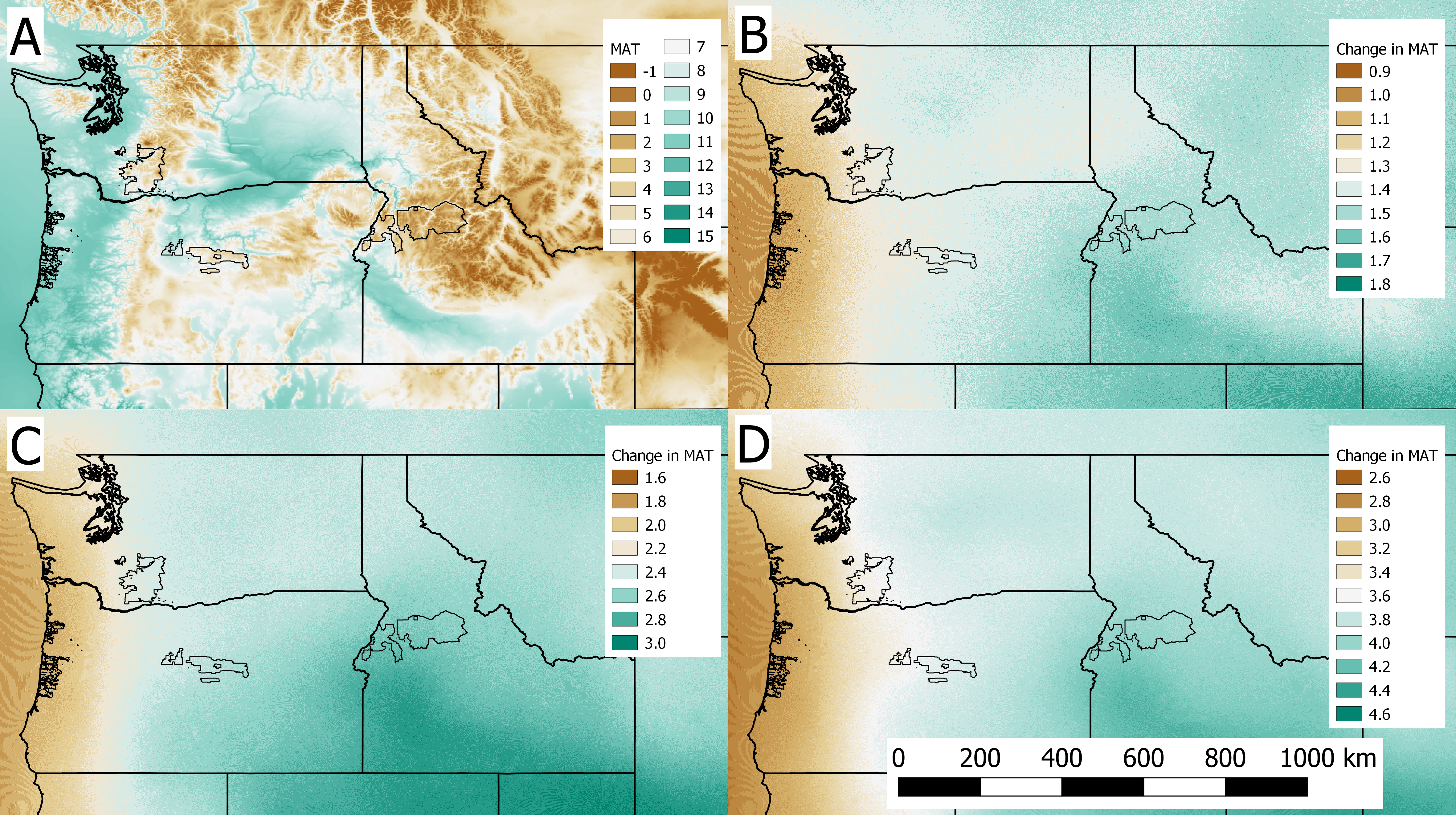


Figure S1. Mean annual temperature (MAT) in °C for (A) northwestern USA in year 1990, and (B) estimated change in MAT used by Climate-FVS under the Ensemble 6.0 climate scenario for years between 1990 and 2030, (C) between 1990 and 2060, and (D) between 1990 and 2090 used by the future climate scenario Ensemble 6.0. Data downloaded from the Climate-FVS server [1].


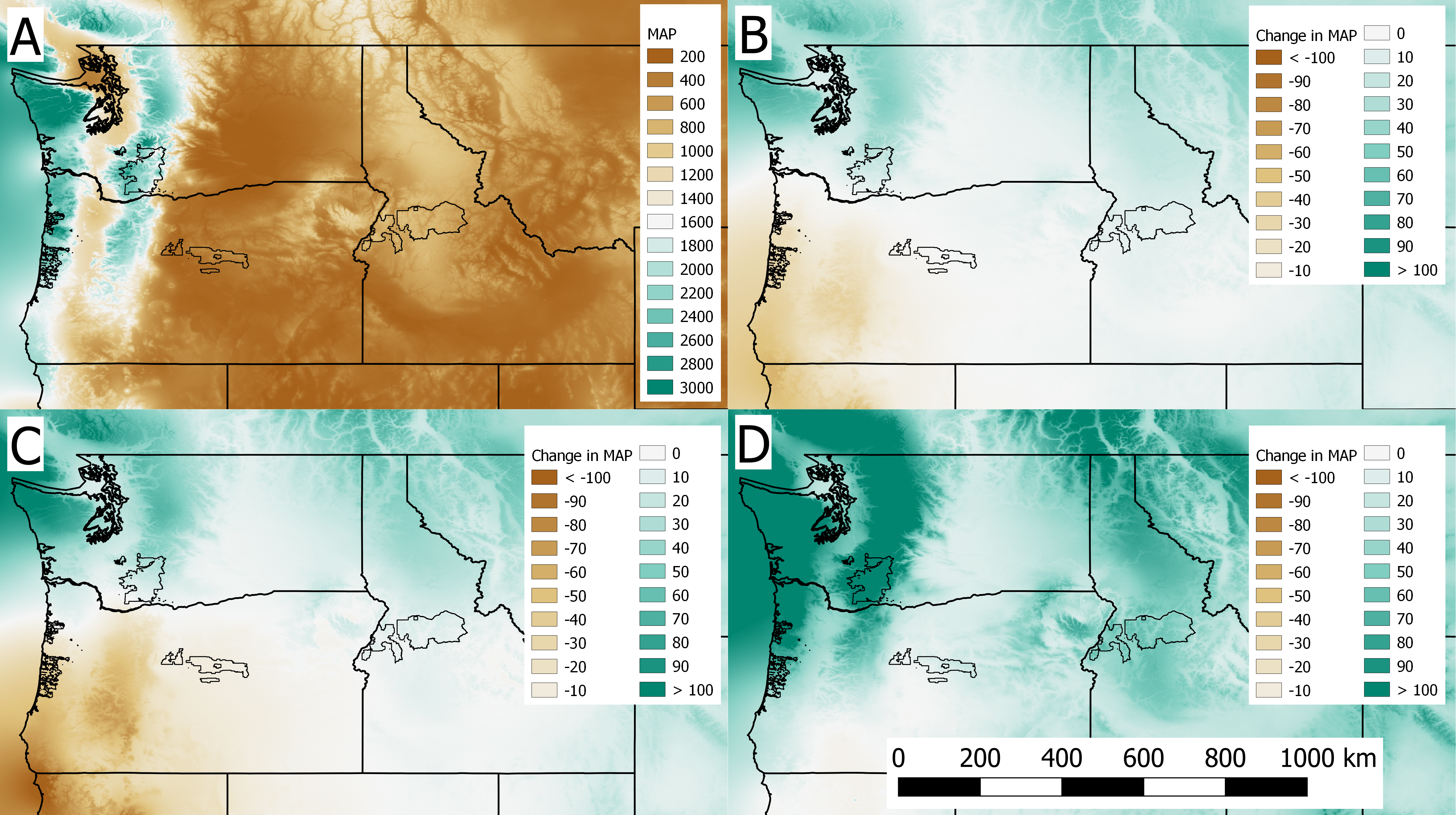


Figure S2. Mean annual precipitation (MAP) in mm for (A) northwestern USA in year 1990, and (B) estimated change in MAP used by Climate-FVS under the Ensemble 6.0 climate scenario for years between 1990 and 2030, (C) between 1990 and 2060, and (D) between 1990 and 2090 used by the future climate scenario Ensemble 6.0. Data downloaded from the Climate-FVS server [1].


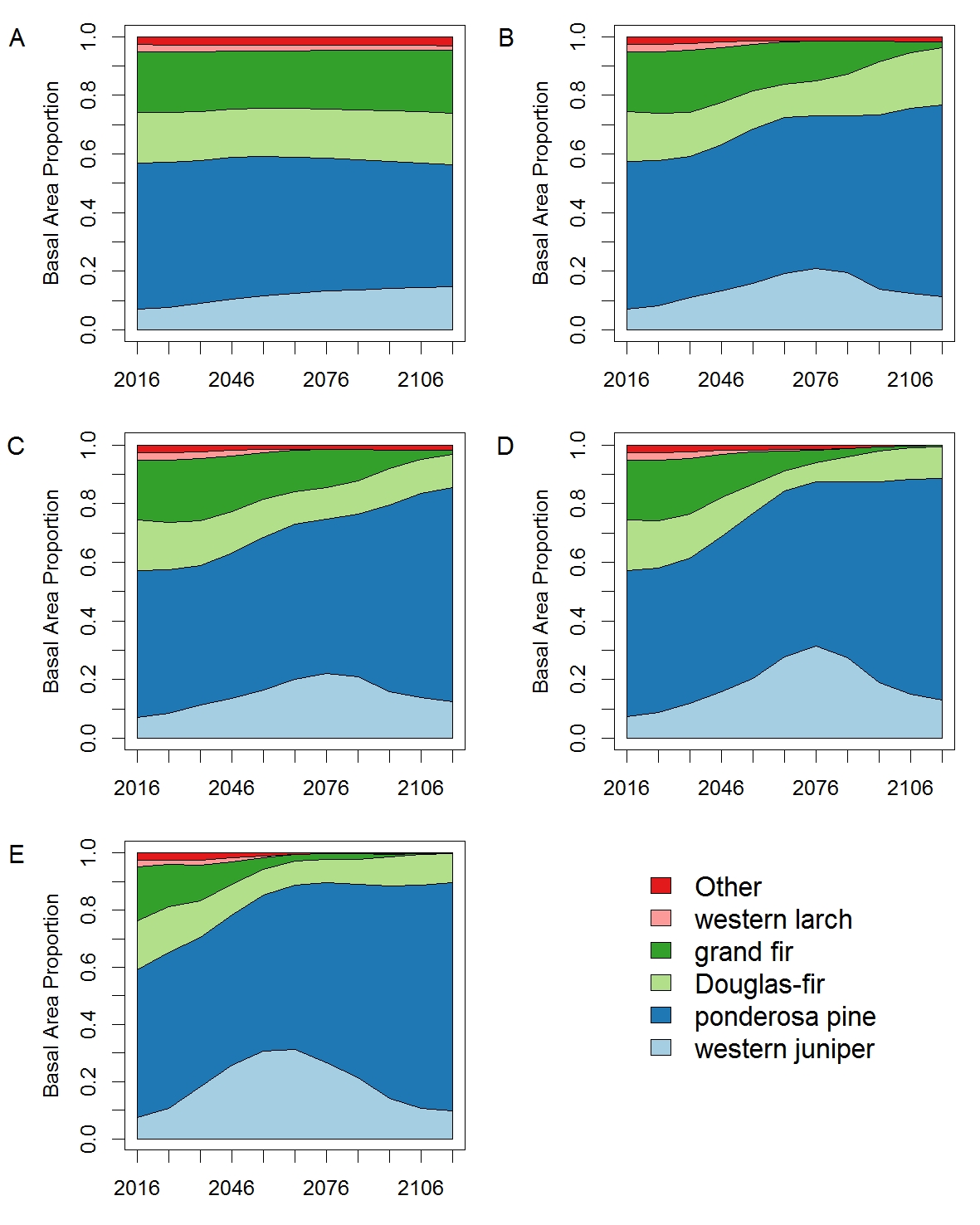


Figure S3: Basal area proportions for select tree species on the Ochoco National Forest simulated with the base disturbance level and: (A) no climate change, (B) dClim Off, (C) dClim 2.0, (D) dClim 1.0 (default setting), and (E) dClim 0.5.


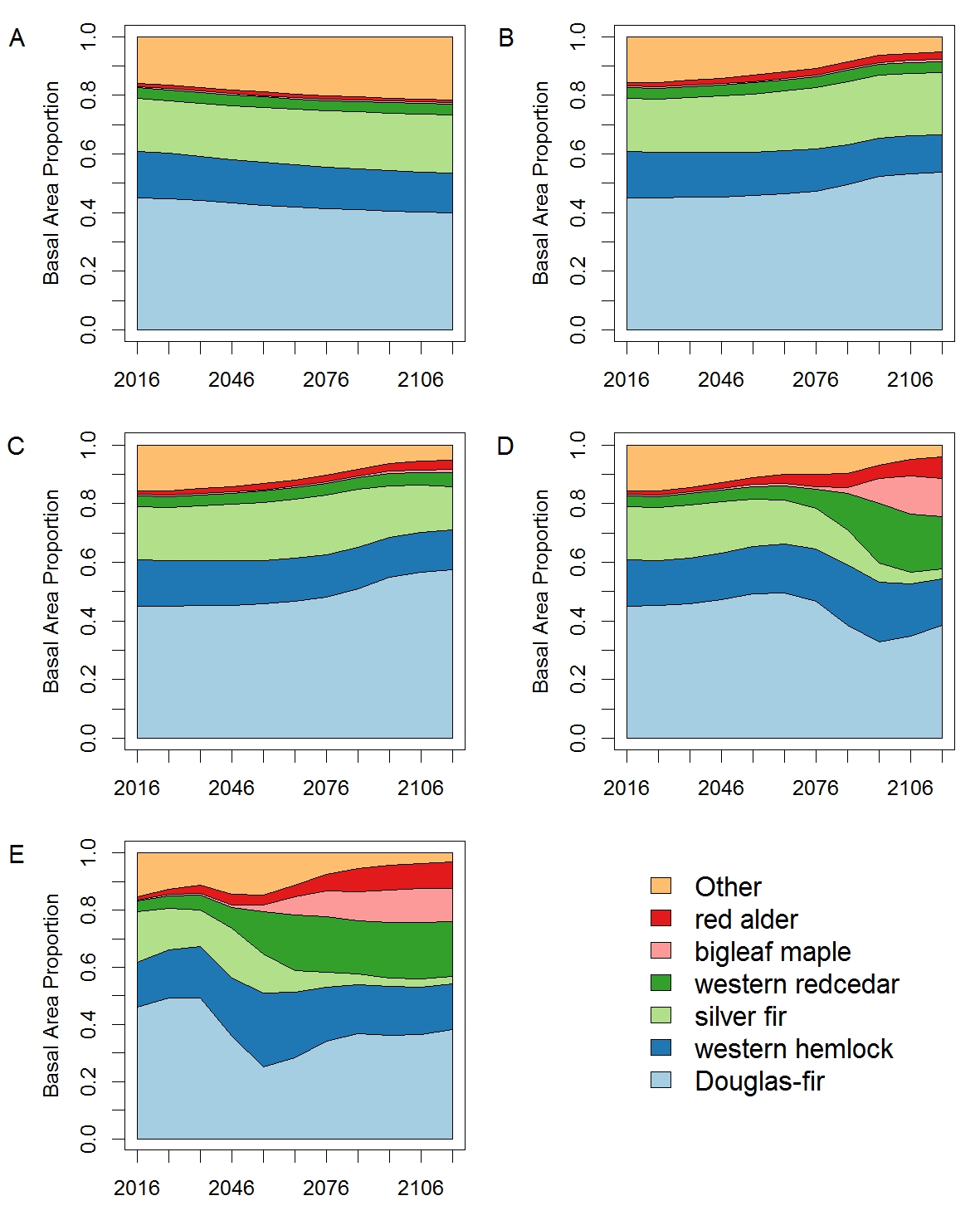


Figure S4: Basal area proportions for select tree species on the Gifford Pinchot National Forest simulated with the base disturbance level and: (A) no climate change, (B) dClim Off, (C) dClim 2.0, (D) dClim 1.0 (default setting), and (E) dClim 0.5.


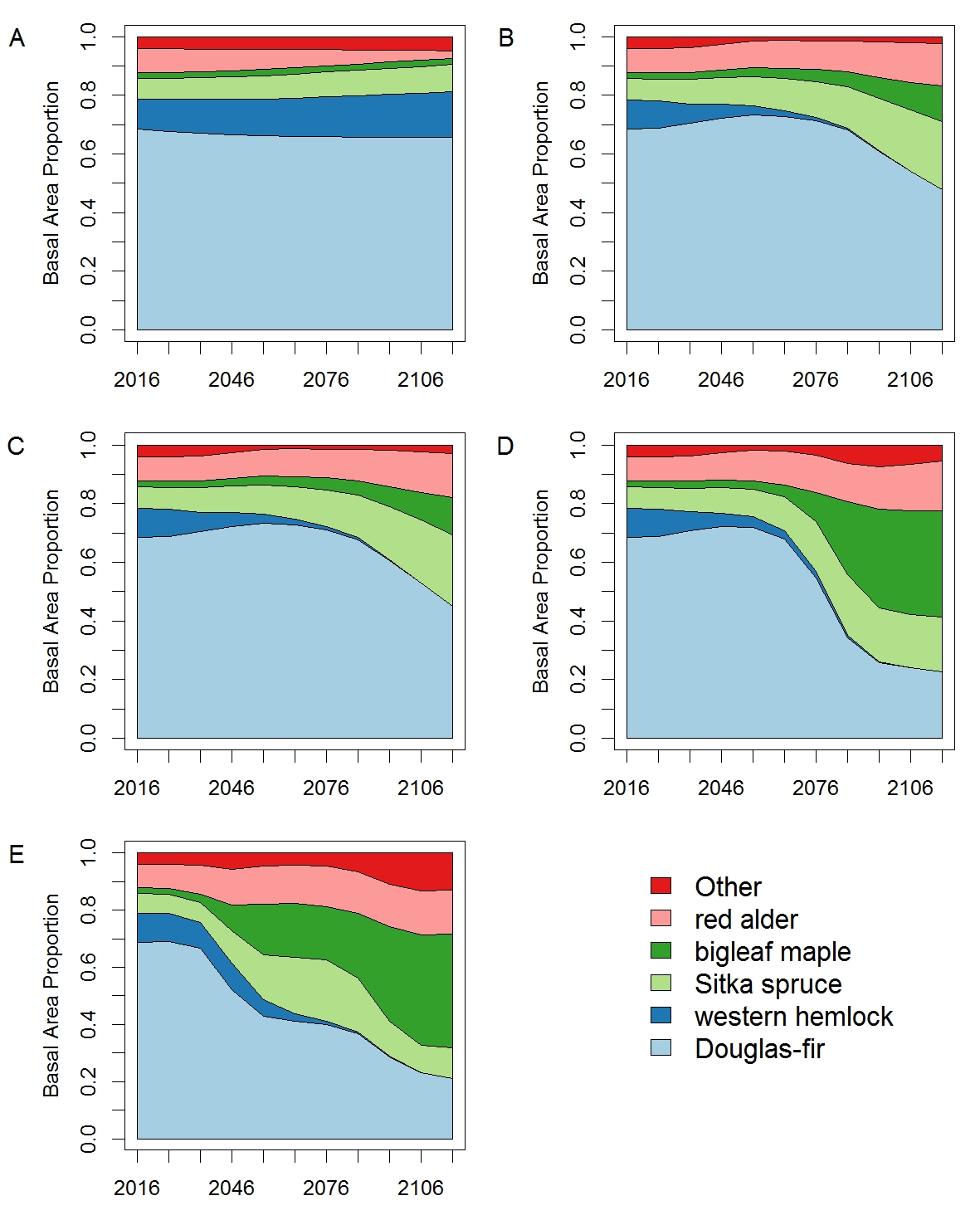


Figure S5: Basal area proportions for select tree species on the Siuslaw National Forest simulated with the base disturbance level and: (A) no climate change, (B) dClim Off, (E) dClim 2.0, (D) dClim 1.0 (default setting), and (E) dClim 0.5.


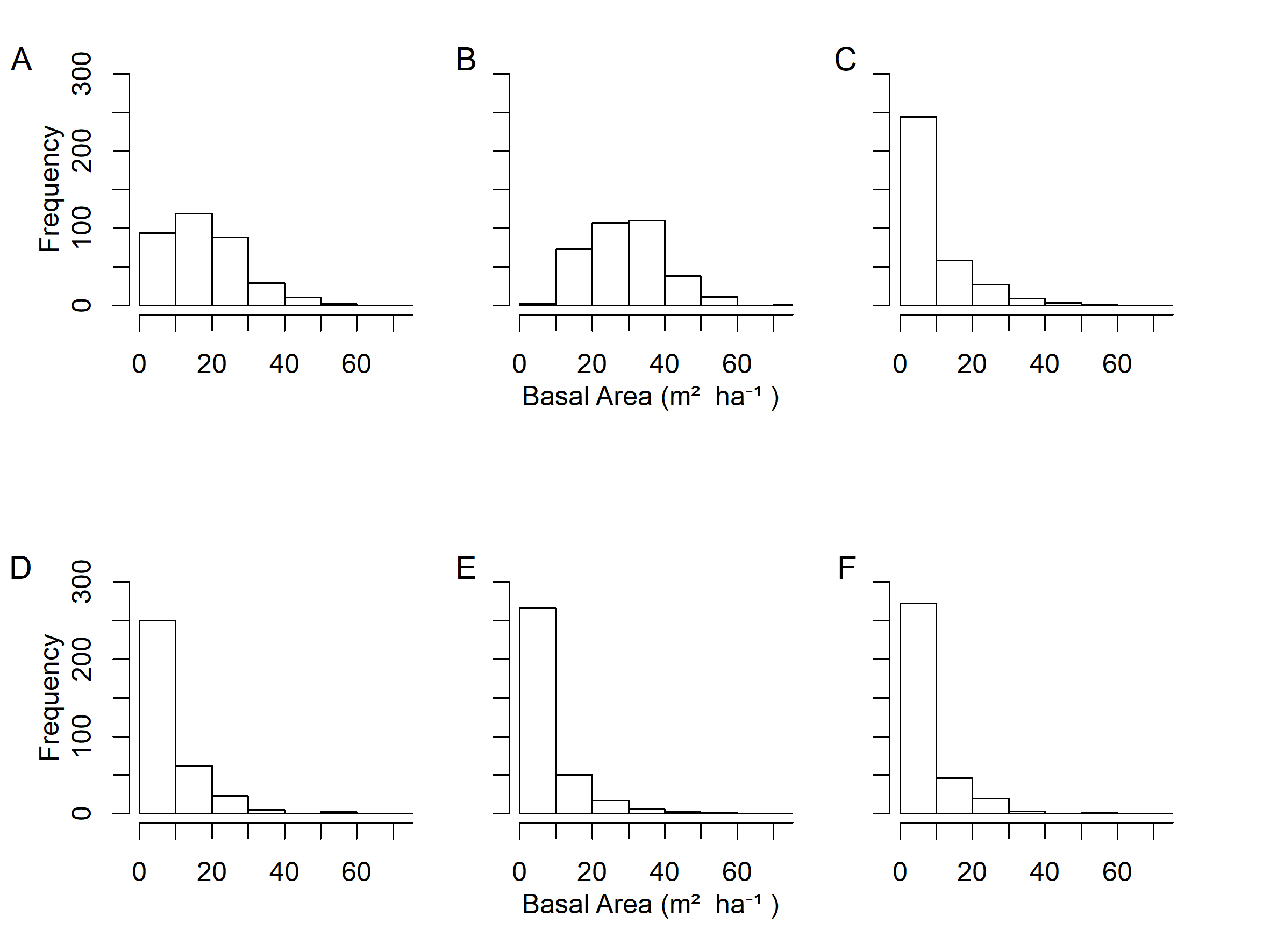


Figure S6: Ochoco National Forest plot-level basal area distribution simulated using the base disturbance level for (A) current conditions (year=2016); (B) future conditions under no climate change (year=2116); (C) future conditions under dClim 2.0 (year=2116); (D) future conditions under dClim Off (year=2116); (E) future conditions under dClim 1.0 (year=2116); (F) future conditions under dClim 0.5 (year=2116).


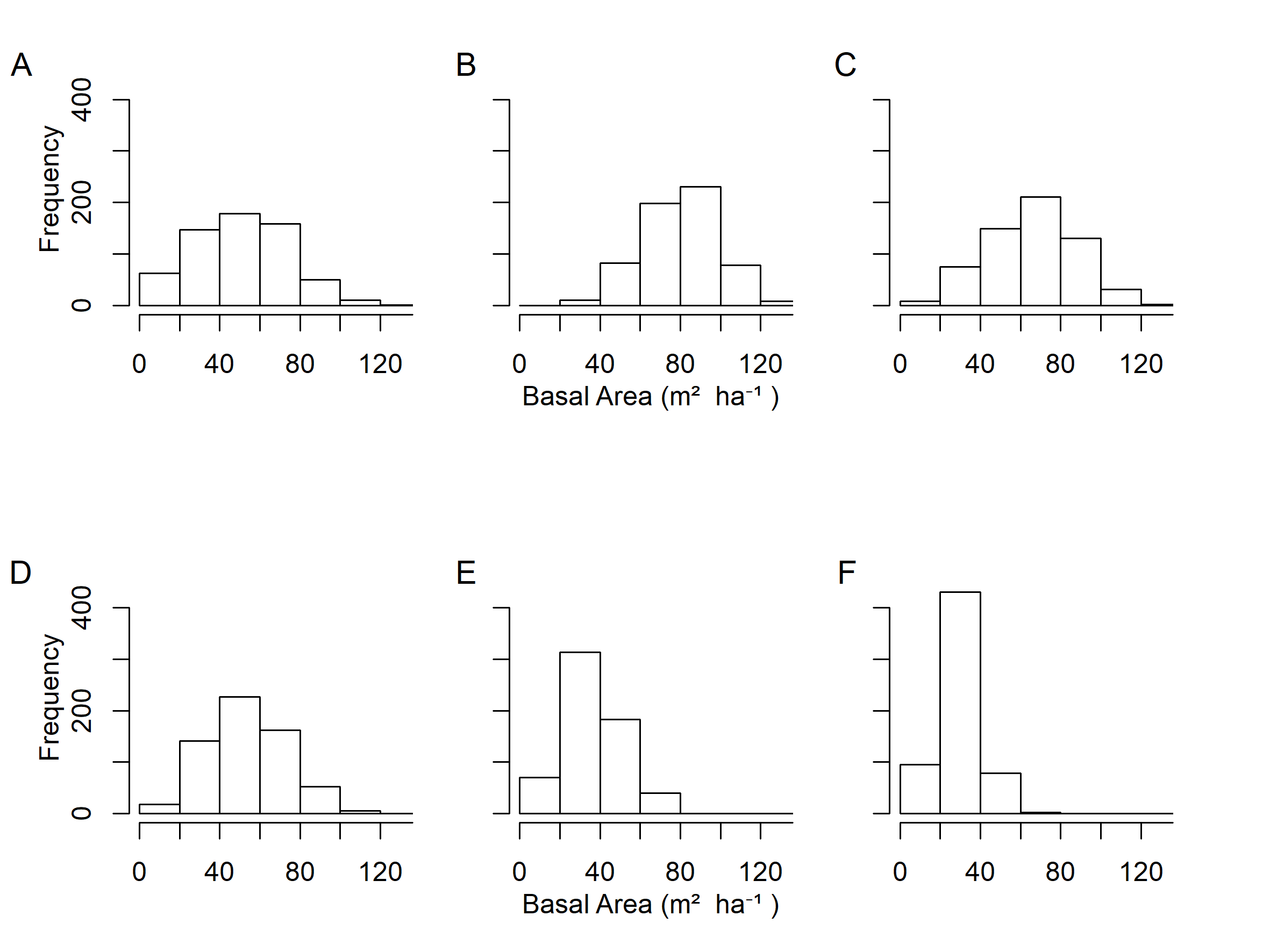


Figure S7: Gifford Pinchot National Forest plot-level basal area distribution simulated using the base disturbance level for (A) current conditions (year=2016); (B) future conditions under no climate change (year=2116); (C) future conditions under dClim 2.0 (year=2116); (D) future conditions under dClim Off (year=2116); (E) future conditions under dClim 1.0 (year=2116); (F) future conditions under dClim 0.5 (year=2116).


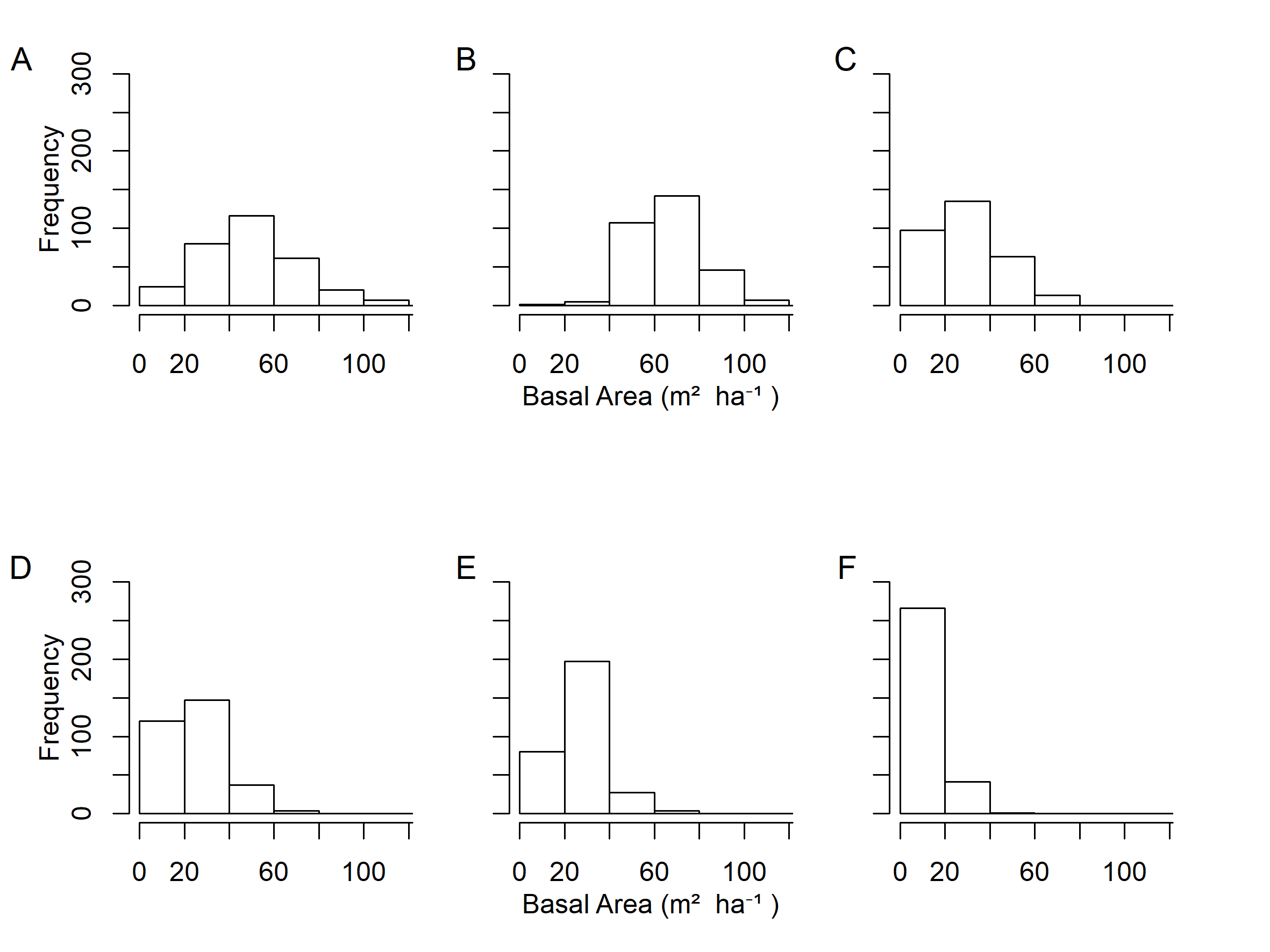


Figure S8: Siuslaw National Forest plot-level basal area distribution simulated using the base disturbance level for (A) current conditions (year=2016); (B) future conditions under no climate change (year=2116); (C) future conditions under dClim 2.0 (year=2116); (D) future conditions under dClim Off (year=2116); (E) future conditions under dClim 1.0 (year=2116); (F) future conditions under dClim 0.5 (year=2116).

**References**

1. Climate-FVS. Climate Estimates and Plant-Climate Relationships. 2019. Available from: http://charcoal.cnre.vt.edu/climate/customData/fvs_data.php. Accessed 3 Mar 2019.
